# Treating Thyroid Associated Ophthalmopathy in Pediatric Patients

**DOI:** 10.3389/fendo.2022.900204

**Published:** 2022-06-28

**Authors:** Tianyu Dong, Zhujun Fu, Xu Wang

**Affiliations:** ^1^ TriApex Laboratories Co., Ltd., Nanjing, China; ^2^ Department of Ophthalmology, Children’s Hospital of Nanjing Medical University, Nanjing, China; ^3^ Department of Endocrinology, Children’s Hospital of Nanjing Medical University, Nanjing, China

**Keywords:** thyroid associated ophthalmopathy, pediatrics, IGF-1R, teprotumumab, children

## Abstract

Thyroid associated ophthalmopathy (TAO) is a common extra-thyroid clinical manifestation of Graves’ disease. It is an inflammatory disease of the eye and orbital tissues. Up to one-third of pediatric Graves’ disease patients could be diagnosed with TAO. The symptoms can be variable with remissions and exacerbations of pediatric Graves’ disease, which has negative effects on the quality of life in children. Teprotumumab is a fully human IgG1κ type monoclonal antibody targeting insulin-like growth factor-1 receptor (IGF-1R), and was approved for the treatment of TAO as a “breakthrough therapy” by the FDA in 2020. Nevertheless, the safety and effectiveness have not been established in pediatric patients. IGF-1R plays an important role in human development, which raises concerns of developmental toxicity. As presented in the pharmacology review report, juvenile monkeys were tested in two separate repeated-dose toxicity studies and no NOAEL was identified. Teprotumumab affected the growth, thymus, spleen and decreased the bone growth. Younger animals seemed to be more sensitive to the effects on normal growth and normal thymus. Hearing impairment posed additional risk to the potential pediatric use, especially for school-age children. Considering the nature of the target, Teprotumumab should not be used empirically in children. More efforts would be made for the further development of teprotumumab for pediatric use.

## Introduction

Either adult or child patients with Graves’ disease could suffer from the ophthalmic thyroid associated ophthalmopathy (TAO), but obviously few attentions were paid for the latter population. Up to one-third of pediatric Graves’ disease patients could be diagnosed with TAO (1). The symptoms can be variable with remissions and exacerbations of primary disease (2), which seriously affects the quality of life. Currently, glucocorticoid is the first-line therapy for pediatric TAO, but the efficacy was controversial and there could be risks for severe adverse reactions (3, 4). Activation of insulin-like growth factor-1 receptor (IGF-1R) signaling and overexpression of IGF-1R in the orbital fibroblasts, B cells and T cells were reported in Graves’ disease patients (5). Teprotumumab is a fully human IgG1κ type monoclonal antibody targeting IGF-1R, and is originally indicated for cancer treatment. The antibody specifically binds to IGF-1R and blocks its activation and signaling. The drug was approved for the treatment of TAO as a “breakthrough therapy” by the FDA in 2020. Nevertheless, the safety and effectiveness have not been established in pediatric patients.

## Concerns About Using Teprotumumab in Children

Considering the nature of the target, teprotumumab should not be used empirically in children. Despite of the proven elevation of IGF-1R in adult TAO patients, pediatric data are scarce. Whether there might be any differences in the etiology involved IGF-1R on TAO between adults and children are largely unknown. Besides, IGF-1R plays an important role in human development (6), which raises concerns of developmental toxicity. Actually, as presented in the pharmacology review report, juvenile monkeys were tested in two separate repeated-dose toxicity studies (both 13 weeks) and no NOAEL (no observed adverse effect level) was identified (7). Teprotumumab affected the growth, thymus, spleen and decreased the bone growth. Younger animals seemed to be more sensitive to the effects on normal growth and normal thymus. Moreover, literature has also documented the functions of IGF-1R in the developmental reproduction and central nervous systems (8–10). Although it is not clear whether teprotumumab could affect these systems, attention can be paid in future studies. Other common adverse actions of teprotumumab indicated for TAO include infusion reactions, exacerbation of preexisting inflammatory bowel disease, muscle spasm, nausea, alopecia, diarrhea, fatigue, hyperglycemia, hearing impairment, dry skin, dysgeusia and headache (11). Most of which are considered manageable based on monitoring and intervention. Recently, there are increasingly case reports on the ototoxicity of teprotumumab (12–14). Even though current data indicate that most of the otologic symptoms resolved after treatment, hearing loss may be persistent in adults (15). Hearing impairment posed additional risk to the potential pediatric use, especially for school-age children.

## Discussion

Observational studies exploring the expression of IGF-1 and IGF-1R in children with TAO may help to verify the therapeutic potential of teprotumumab. Further toxicity studies using juvenile animals are particularly important for exploring the developmental effects and obtaining toxicological threshold doses (e.g. NOAEL) in order to set the basis for the design of initial dose for pediatric use. Mechanism studies could be conducted to confirm the clinical significance when necessary. If neither safe doses nor acceptable developmental toxicity might be observed in additional juvenile toxicology studies, it would be recommended to find out the youngest age for a pediatric TAO patient to receive teprotumumab therapy safely based on all available data. Changing the current route of administration of teprotumumab (iv) to topical administration (e.g. periorbital injection) may be an alternative to reducing systemic toxicity. Clinical trials are essential and an evidence-based risk-benefit assessment should be performed. If the results cannot reach the endpoints, more efforts would have to be made to discover new targets and develop novel medicines for treating TAO in pediatric patients.

## Data Availability Statement

The original contributions presented in the study are included in the article/supplementary material. Further inquiries can be directed to the corresponding author.

## Author Contributions

XW contributed to the conception of the work and reviewed the manuscript; TD drafted and reviewed the manuscript; ZF made critical revisions to the manuscript. All authors contributed to the article and approved the submitted version.

## Conflict of Interest

Author TD is employed by TriApex Laboratories Co., Ltd.

The remaining authors declare that the research was conducted in the absence of any commercial or financial relationships that could be construed as a potential conflict of interest.

## Publisher’s Note

All claims expressed in this article are solely those of the authors and do not necessarily represent those of their affiliated organizations, or those of the publisher, the editors and the reviewers. Any product that may be evaluated in this article, or claim that may be made by its manufacturer, is not guaranteed or endorsed by the publisher.
